# Potential targets and molecular mechanism of miR-331-3p in hepatocellular carcinoma identified by weighted gene coexpression network analysis

**DOI:** 10.1042/BSR20200124

**Published:** 2020-06-25

**Authors:** Qingjia Chi, Xinge Geng, Kang Xu, Chunli Wang, Han Zhao

**Affiliations:** 1 Department of Mechanics and Engineering Structure, Wuhan University of Technology, Wuhan 430070, China; 2 Department of Cardiovascular Surgery, Union Hospital, Tongji Medical College, Huazhong University of Science and Technology, Wuhan 430030, China; 3 ‘111’ Project Laboratory of Biomechanics and Tissue Repair, Bioengineering College, Chongqing University, Chongqing 400044, China; 4 Institute of Biomedical Sciences, School of Medicine, Jianghan University, Wuhan 430056, China

**Keywords:** GEO, miR-331-3p, TCGA, WGCNA

## Abstract

Hepatocellular carcinoma (HCC) is one of the most common malignant tumor. miR-331-3p has been reported relevant to the progression of HCC, but the molecular mechanism of its regulation is still unclear. In the study, we comprehensively studied the role of miR-331-3p in HCC through weighted gene coexpression network analysis (WGCNA) based on The Cancer Genome Atlas (TCGA), Gene Expression Omnibus (GEO) and Oncomine. WGCNA was applied to build gene co-expression networks to examine the correlation between gene sets and clinical characteristics, and to identify potential biomarkers. Five hundred one target genes of miR-331-3p were obtained by overlapping differentially expressed genes (DEGs) from the TCGA database and target genes predicted by miRWalk. The critical turquoise module and its eight key genes were screened by WGCNA. Enrichment analysis was implemented based on the genes in the turquoise module. Moreover, 48 genes with a high degree of connectivity were obtained by protein–protein interaction (PPI) analysis of the genes in the turquoise module. From overlapping genes analyzed by WGCNA and PPI, two hub genes were obtained, namely coatomer protein complex subunit zeta 1 (COPZ1) and elongation factor Tu GTP binding domain containing 2 (EFTUD2). In addition, the expression of both hub genes was also significantly higher in tumor tissues compared with normal tissues, as confirmed by analysis based on TCGA and Oncomine. Both hub genes were correlated with poor prognosis based on TCGA data. Receiver operating characteristic (ROC) curve validated that both hub genes exhibited excellent diagnostic efficiency for normal and tumor tissues.

## Introduction

Liver cancer is the fifth and third malignant tumor with morbidity and mortality [1]. In clinical diagnosis, hepatocellular carcinoma (HCC) cannot easily be diagnosed at an early stage, and is often not detected until the late stage of cancer [2,3]. HCC is the most common type of liver cancer, accounting for 75% of liver cancer [4]. Although some progress has been made in diagnosis and treatment strategies, the high metastasis rate and recurrence rate of HCC make it difficult for patients with advanced HCC to be effectively treated [5,6]. Thus, it is meaningful for the treatment to study the underlying molecular and identify novel markers for diagnosis and prognosis.

MicroRNAs (miRNAs) are a class of short, highly conserved, single-stranded non-coding RNAs, each with a length of 18–25 nucleotides [7,8]. MiRNAs play an important part in a variety of biological processes (BPs) by regulating gene expression post-transcriptionally [9,10]. According to recent studies, Let-7, miR-101 and miR-370 are down-regulated in HCC [11–13]. While miR-155, miR-21, miR- 221, miR-146a, miR-515 and miR-224 are up-regulated in HCC [14–18]. At the same time, these miRNAs have certain diagnostic value and prognostic significance for HCC.

In our previous studies, we combined computational, experimental and bioinformatic methods to investigate the biophysical properties of the nucleic acids [19,20], and the molecular mechanism of inflammation regulation of small molecule drugs [21–23]. miR-331-3p is considered to be an important cancer-related mircoRNA. Chen et al. found that miR-331-3p is an up-regulated micoRNA in pancreatic cancer (PC), while miR-331-3p inhibits suppression of tumorigenicity 7 like (ST7L) and epithelial mesenchymal transition (EMT)-mediated tumor metastasis, thereby promoting PC cell proliferation. miR-331-3p could be used as a potential diagnostic biomarker and drug target [24]. Similarly, Gu et al. found that the recurrence rate of esophageal adenocarcinoma patients with high-expressing serum miR-331-3p was lower, and miR-331-3p could be a potential biomarker for predicting tumor recurrence in patients with esophageal adenocarcinoma [25]. Yang et al. found that miR-331-3p inhibits the development of gastric cancer by targeting MSI 1 and serve as an indicator of gastric cancer prediction and prognosis [26]. Novel targets of miR-331-3p for liver cancer were also revealed. miR-331-3p promotes liver cancer and secondary EMT-mediated metastasis by inhibiting PLPPP-mediated dephosphorylation of protein kinase B (AKT). And miR-331-3p can serve as a new therapeutic target and a potential prognostic biomarker [27]. Similarly, miR-331-3p down-regulates E2F1 to promote the development and metastasis of HCC, indicating the possible application of miR-331-3p in predicting the prognosis and treatment of HCC [28]. There are reports that miR-331-3p expression is affected by viruses in HCC, and hepatitis B virus (HBV) is a typical virus that up-regulates miR-331-3p in HCC cell lines. miR-331-3p reduces von Hippel–Lindau tumor suppressor (VHL) expression by directly targeting its 3′-UT [29].

However, the molecular mechanism of miR-331-3p is not clear in the development of HCC. We aimed to comprehensively studied the role of miR-331-3p in HCC through WGCNA based on TCGA, GEO and Oncomine. WGCNA were applied to build gene co-expression networks to examine the correlation between gene sets and clinical characteristics, and to identify hub genes and critical pathway.

## Materials and methods

**Figure 1:**
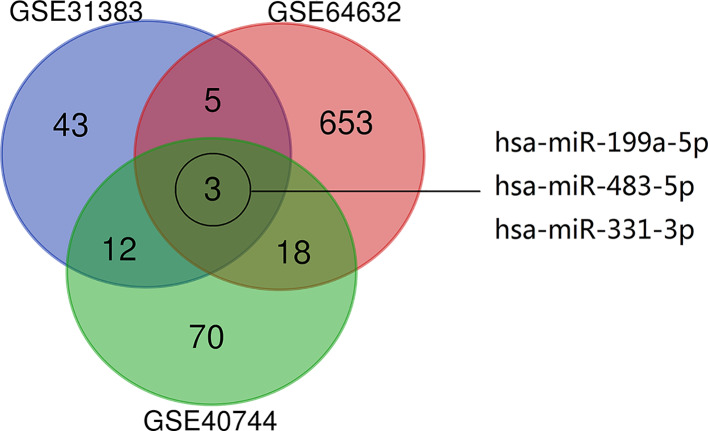
Venn diagram of a differential analysis dataset Datasets (GSE31383, GSE40744, GSE64632) were selected. Non-coding RNA profiling by array method in the dataset was implemented.

Differential analysis is performed on three datasets (GSE31383, GSE40744, GSE64632) in the GEO database. These datasets implemented non-coding RNA profiling by microarray technology. Differentially expressed microRNAs (DEMs) were screened (*P*<0.05, |log FC|>1) individually obtained from these datasets were overlapped to get three DEMs (miR-199a-5p, miR-483-5p, miR-331-3p) (Figure 1). The role of miR-199a-5p [30–34] and miR-483-5p [35–39] in HCC has been extensively studied. However, the molecular mechanism of miR-331-3p in HCC is not clear and remains to be explored. Consequently, miR-331-3p was chosen for further study.

First, the expression of miR-331-3p in HCC was obtained by integrating multiple ways, and the prognostic value analysis and comprehensive meta-analysis of miR-331-3p were performed. Then, the HCC-related differentially expressed genes (DEGs) from TCGA were overlapped with the target genes of miR-331-3p predicted from 12 databases to obtain overlapping target genes. Through WGCNA, the overlapping genes and the key genes in the modules that play an important part in the development of HCC are obtained. Through the gene ontology (GO) enrichment analysis and Kyoto, Encyclopedia of Genes and Genomes (KEGG) pathway analysis of gene sets in important modules to explore its role in the biological process of HCC. Through PPI analysis of the gene sets in the important modules, genes with high gene connectivity are obtained. Hub genes were obtained from key genes and genes with high gene connectivity obtained from PPI analysis. The relationship between the miR-331-3p targeted genes and HCC was explored from the aspects of gene expression and DNA methylation. The working flow chart of the study is shown in Supplementary Figure S1.

### TCGA high-throughput data for HCC patients

The batch download mode was used to download the TCGA data and extract the HCC expression data using the TCGA simple download tool-V16 in SangerBox software (http://sangerbox.com/). TCGA Easy Download Tool-V16 is obtained from the TCGA database website (https://portal.gdc.cancer.gov/; accessed in May 2019). A total of 422 mature miRNA expression profile samples were obtained, including 372 HCC tissue samples and 50 paracancerous tissue samples. Further we use SangerBox to combine the expression data in 422 samples and logarithmic conversion to obtain miRNA expression profiles. Finally, high-throughput data of TCGA of HCC patients with miR-331-3p were obtained from miRNA expression profiles.

### GEO microarray data screening

The research conducted a keyword search of HCC-related microarray data on the GEO database (Gene Expression Omnibus; https://www.ncbi.nlm.nih.gov/gds/; accessed August 2019). For: (malignant * OR cancer OR tumor OR tumor OR neoplasm * OR carcinoma) AND (hepatocellular OR liver OR hepatic OR HCC) AND (microRNA OR miRNA OR ‘micro RNA’ OR ‘small temporal RNA’ OR ‘non coding RNA’ OR ncRNA OR ‘small RNA’). The data retains that meets the following conditions is reserved for further analysis: (1) the data in the dataset are from humans; (2) the dataset has both HCC tissue expression data and healthy or adjacent tissue as control group expression data; (3) the number of samples in the experimental group and the control group is greater than 3; (4) the dataset contains the expression data of miR-331-3p.

### Comprehensive meta-analysis

The study used RevMan 5.3 (London, UK) for a comprehensive meta-analysis. Standard mean difference (SMD) and 95% confidence interval (CI) were used to measure continuous results. We use the Mantel–Haenszel formula (fixed effects model) or DerSimonian–Laird formula (random effects model) to summarize SMDs and perform Cochrane's *Q* test (Chi-square test; Chi^2^) and inconsistency (*I*^2^) tests to assess heterogeneity. A random effect model is applied as heterogeneity is shown (*P* <0.05 or *I*^2^>50%). Otherwise, a fixed-effect model is selected. A funnel plot of Egger's test was used to assess publication bias. A significant asymmetry of the funnel plot was determined when *P*<0.1.

### Prognostic value of miR-331-3p

Starbase v3.0 (http://starbase.sysu.edu.cn/index.php) is an open source platform that combines miRNA expression data and prognosis data with various cancers. We determined whether miR-331-3p is an effective prognostic biomarker for HCC by comparing the overall survival (OS) of HCC patients with different expression levels of miR-331-3p.

### Target gene prediction of miR-331-3p

GEPIA (http://gepia.cancer-pku.cn/detail.php) is an interactive web server that uses standard processing flow of TCGA and GTEx projects to analyze gene expression data, which contains 9736 tumor and 8587 normal samples. We used GEPIA to access DEGs (*P*<0.05 and |logFC|>1) between HCC and paracancerous tissues using its embedded Limma package. There are 12 databases (Microt4, miRWalk, mir-bridge, miRanda, miRDB, miRMap, Pictar2, PITA, MiRNAMap, RNAhybrid, RNA22 and Targetscan) in miRWalk2.0 version (http://zmf.umm.uni-heidelberg.de/apps/zmf/mirwalk2/) to predict target genes of miR-331-3p. At least genes coexisted in five database were identified as the DEGs. Then, the DEGs obtained by GEPIA and the target genes predicted by miRWalk2.0 were compared to obtain overlapping genes of the two.

### WGCNA explores important modules and key genes of overlapping genes

WGCNA is a systematic biology method to describe the pattern of gene correlation between different samples. It can classify highly synergistically changing gene sets based on the interconnectivity of gene sets and the relationship between gene sets and phenotypes. Associations identify candidate biomarker genes or therapeutic targets. Specific steps are as follows.

#### Importing data and preprocessing

Based on the overlapping gene set obtained above, by matching HCC-related RNA-seq data and clinical data in the TCGA database, a standardized transcript expression profile of the overlapping genes and clinical phenotypic data of HCC are obtained. The expression profile matrix and phenotypic matrix of overlapping genes were imported into R script, and then the expression profile matrix was clustered to remove outliers and genes.

#### Define gene expression similarity matrix

Based on the expression profile matrices of overlapping genes, a gene expression similarity matrix *S* of overlapping genes is calculated.

$$S=[S_{XY}]=[|cor(X,Y)|]=\left[\left|\frac{\sum_{n}\left(X_{i}-E(X)\right)(Y_{i}-E(Y))}{\sqrt{\sum_{n}{\left(X_{i}-E(X)\right)}^{2}}\sqrt{\sum_{n}{\left(Y_{i}-E(Y)\right)}^{2}}}\right|\right]$$

Among them, *S_XY_* is the similarity between genes *X* and *Y*, the absolute value of the Pearson correlation coefficient of vectors *X* and *Y*; *X* is the expression vector of gene *X*; *Y* is the expression vector of gene *Y*; *n* is the number of samples; *E (X)* and *E (Y)* represent the mean of the vectors *X* and *Y*, respectively.

#### Compute adjacency matrix

Select the exponential weighting coefficient *β*, and the selection of *β* should satisfy the law of scale-free networks. The similarity matrix *S* is further transformed into an adjacency matrix *A*.

$$A=\left[a_{XY}\right]=\left[|S_{XY}{|}^{\beta }\right],$$

where *a_XY_* shows the adjacency coefficients of genes *X* and *Y*.

#### Create topological overlap matrix

Based on the adjacency matrix *A*, a topological overlap matrix TOM is constructed.

$$\text{TOM}=\left[\omega_{XY}\right]=\left[\frac{l_{XY}+a_{XY}}{\min\left\{k_{X},k_{\text{Y}}\right\}+1-a_{XY}}\right]$$

$$\begin{array}{ccc} l_{XY}=\sum_{u}a_{Xu}a_{uY} & k_{X}=\sum_{u}a_{Xu} & k_{Y}=\sum_{u}a_{Yu} \end{array}$$

The *u* in *l_XY_* indicates the set of genes adjacent to the genes *X* and *Y* at the same time; *k_X_* and *k_Y_* the indicate the set of genes adjacent to genes *X* and *Y*, respectively.

### Building a systematic clustering tree

Calculate the node dissimilarity $d_{XY}^{\omega }$ and construct a node dissimilarity matrix *dissTOM*. Based on the matrix *dissTOM*, a dynamic hybrid cutting algorithm is used to identify network modules from the system cluster tree.

$$dissTOM=\left[d_{XY}^{\omega }\right]=\left[1-\omega_{XY}\right]$$

$d_{XY}^{\omega }$ indicates the degree of dissimilarity between genes *X* and *Y*.

### Map gene co-expression networks

Eigenvector genes (module eigengene, ME) of each module is calculated. ME means the overall expression level of the module. Pearson coefficients between modules ME were calculated, and the module ME was clustered using average-linkage hierarchical clustering method. The modules with higher similarity were combined to obtain a co-expression network.

### Screening gene modules

Correlate the expression level of the module with the phenotypic data, and calculate the Pearson correlation coefficient $MT_{M}^{T}$ between each module's ME and the sample trait feature vector. Select modules that significantly associated with phenotypic data for downstream analysis. When *P*<0.05, results are considered statistically significant.

$$MT_{M}^{T}=cor(ME^{M},ST^{T})$$

Among them, *ME^M^* indicates the ME vector of the *M*th module; *ST^T^* indicates the ST vector of the *T*th personality; and $MT_{M}^{T}$indicates the correlation between the *M*th module and the *T*th personality.

### Identifying pivot genes

The concept of module membership (MM) is introduced to measure the importance of genes in modules. At the same time, gene significance (GS) is introduced to reflect the degree of association between genes and traits. Key genes are identified using screening conditions of MM>0.8 and GS>0.2.

$$MM^{M}(X)=cor(X,ME^{M})$$

$$GS^{T}(X)=cor(X,ST^{T})$$

*MM^M^*(*X*) indicates the significance of the gene *X* in the *M*th module; and *GS^T^* (*X*) indicates the degree of the gene *X*'s prominence in the *T*th module.

### GO enrichment and KEGG pathway analysis

Based on key genes obtained from WGCNA, GO enrichment and KEGG pathway analysis were performed using DAVID 6.8 (https://david.ncifcrf.gov/). *P*<0.05 is considered to be the criteria of statistical significance in the analysis of GO and KEGG pathway enrichment. Three entries in GO enrichment analysis, namely cell component (CC), biological process (BP) and molecular function (MF), reflect the functional annotation of key genes. Critical pathways involved in the activity of miR-331-3p in HCC were analyzed through KEGG pathway analysis.

### PPI network analysis

STRING (https://string-db.org/) is a web server for interactive gene search to generate PPI network. Then the PPI network of key genes collected from WGCNA is obtained from STRING. The nodes and lines in the network graph represent the target genes and their interactions, respectively. For accurate results, nodes in the network with an interaction score of less than 0.4 and nodes not connected to the main network will be deleted. Furthermore, interaction results obtained from STRING were imported into Cytoscape 3.6.1 for visualization and determination of gene connectivity. Gene connectivity is a quantitative indicator to assess the degree of interaction between genes.

### Identification and validation of hub genes

The key genes of the important modules obtained by WGCNA are intersected with genes with a connectivity of ≥8 obtained by PPI network analysis. The hub gene with the highest correlation with HCC among miR-331-3p was obtained. UALCAN (http://ualcan.path.uab.edu) is an interactive web server with TCGA's RNA-seq data and clinical data for 31 cancer types. It can obtain the expression of a single gene in cancer and its survival curve. Use the TCGA simple download tool-V16 in SangerBox software to download the TCGA data and extract the data of coatomer protein complex subunit zeta 1 (COPZ1) and elongation factor Tu GTP binding domain containing 2 (EFTUD2). SPSS was used to draw the ROC curves of COPZ1 and EFTUD2, respectively. The ROC curve was used to distinguish expression data between HCC tissue and control tissue. The area under curve (AUC) value was used to evaluate the clinical diagnostic value of miR-331-3p in HCC. A larger AUC value indicates the higher diagnostic performance. Oncomine (www.oncomine.org) is currently the largest oncogene chip database and comprehensive data mining platform, with 715 gene expression data collections and 86733 samples of cancer and normal tissues. Oncomine can analyze the differential expression of different genes for universal cancer types and their normal tissues. We differentiated target genes by using Oncomine. Differential analysis results of HCC in different data sets were obtained, and the results with significant research significance were selected (*P*<0.05). DiseaseMeth 2.0 (http://bioinfo.hrbmu.edu.cn/diseasemeth/) is the largest database of DNA methylation status today. We use the methylation level of the hub gene obtained in this site in HCC and normal tissues adjacent to the cancer.

## Results

### High-throughput data for TCGA in HCC patients

**Figure 2:**
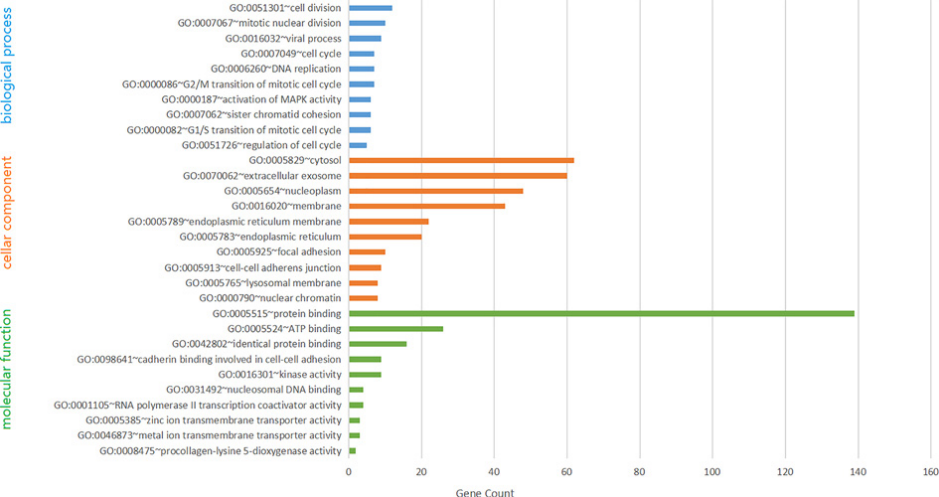
MiR-331-3p expression in HCC and normal tissues in the GEO database MiR-331-3p expression data in HCC and normal tissues were integrated from the datasets (GSE31383, GSE40744, GSE64632), obtained from the GEO database.

Box plots were drawn from the data in the three datasets (GSE31383, GSE64632, GSE40744) of miR-331-3p differential expression obtained above. The expression of miR-331-3p is higher in cancer tissues than in normal tissues (Figure 2).

### GEO data screening

According to the method mentioned above, a keyword search is performed in the GEO database. Save the datasets that meet the requirements and get seven data sets (GSE10694, GSE31383, GSE40744, GSE64632, GSE64989, GSE67882, GSE98269), as shown in Table 1.

**Table 1:** Basic information of the data set studied

Series	Country	Experiment type	Platforms	HCC samples	Healthy samples
GSE10694	China	Non-coding RNA profiling by array	GPL6542	78	88
GSE31383	USA	Non-coding RNA profiling by array	GPL10122	9	10
GSE40744	USA	Non-coding RNA profiling by array	GPL14613	9	7
GSE64632	USA	Non-coding RNA profiling by array	GPL18116	3	3
GSE64989	Germany	Non-coding RNA profiling by array	GPL16384	8	10
GSE67882	India	Non-coding RNA profiling by array	GPL10850	4	8
GSE98269	China	Non-coding RNA profiling by array	GPL20712	3	3
TCGA		miR-seq	Illumina	372	50

Qualified datasets were found from the GEO database and the TCGA database, respectively. (1) The data in the dataset were from humans. (2) The dataset contained HCC tissue expression data and healthy or adjacent tissue control group expression data. The number of samples in both the experimental group and the control group was greater than 3. (4) The data set contained the expression data of miR-331-3p.

### Comprehensive meta-analysis

**Figure 3:**
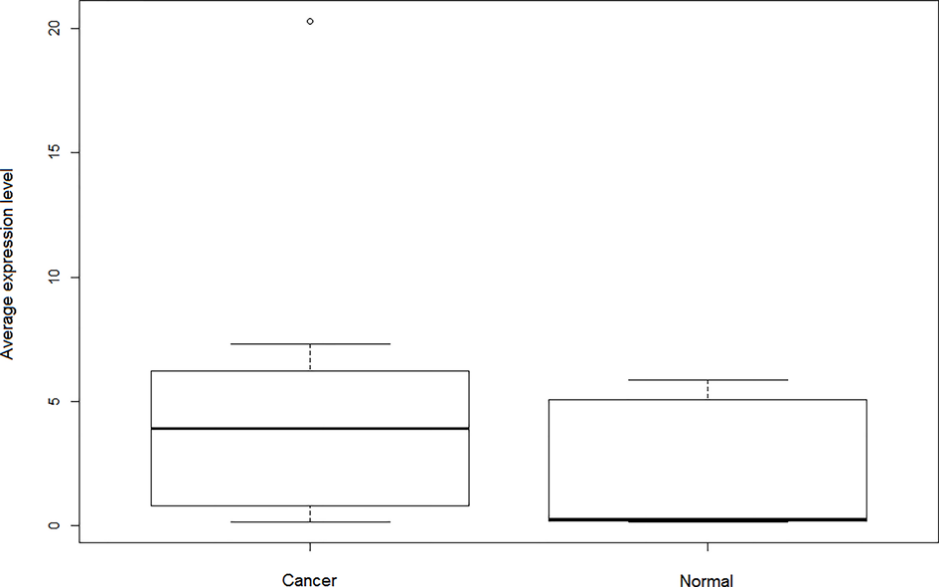
The study performed meta-analysis using RevMan based on data from the GEO and TCGA (**A**) Meta-analysis of data and corresponding forest plots showed no significant difference between HCC and the control group (SMD = 0.26; 95% CI: 0.17–0.36; *P*<0.00001). The random effects model has significant heterogeneity (*P*<0.00001; *I*^2^ = 91%). (**B**) Funnel plot without publication bias.

As mentioned previously, RevMan 5.3 software was used to perform a meta-analysis on the included GEO dataset and TCGA dataset, which included 486 HCC and 179 non-cancerous liver tissues. No momentous difference was found between HCC and the control group (SMD = 0.26; 95% CI: 0.17–0.36; *P*<0.00001). The random effects model has significant heterogeneity (*P*<0.00001; *I*^2^ = 91%). The funnel plot does not indicate publication bias. Figure 3 shows the results of the forest plot and funnel plot.

### Prognostic significance of miR-331-3p

**Figure 4:**
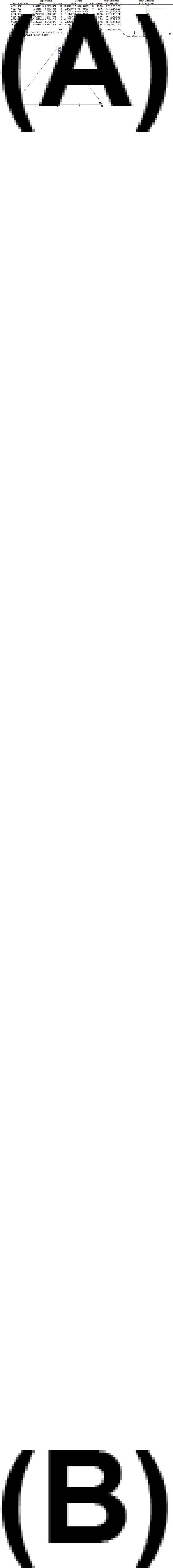
Survival curve of miR-331-3p against HCC based on TCGA data using StarBase v3.0 (median = 21.8, *P* = 0.0015, hazard ratio = 1.77)

Based on the results of StarBase v3.0, the curve of the OS rate of HCC patients with high and low expression of miR-331-3p (Figure 4) found that miR-331-3p has a significant prognostic significance for HCC. OS and enrollment time of the patients are demonstrated in Supplementary Table S1.

### miR-331-3p target gene prediction

The first step is to analyze the genetic high-throughput data of the TCGA database through GEPIA. 2206 HCC-related genes were screened out within the difference threshold (*q*<0.01 and |log2FC|>1). Next, we use miRwalk2.0 to predict the potential target genes and retain the genes found in at least three databases. Six thousand two hundred seventy three potential target genes were obtained. In the third step, the genes obtained in the first two steps are overlapped to obtain 501 overlapping genes (Supplementary Figure S2).

### WGCNA explores important modules and key genes of overlapping genes

**Figure 5:**
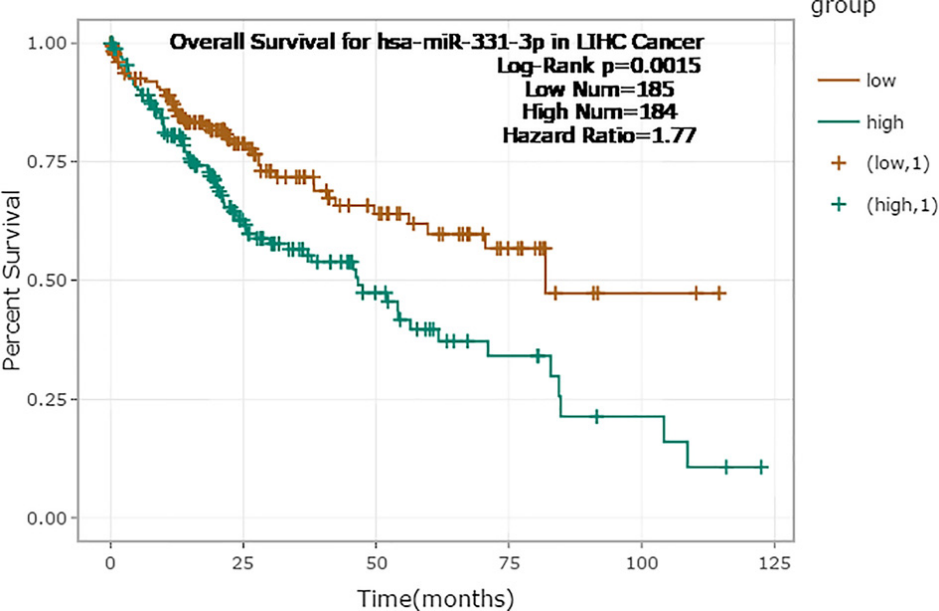
Exploring important modules related to target genes and clinical features through WGCNA (**A**) Analyze the scale-free fitting index (left) and average connectivity (right) of various soft threshold weights. (**B**) Treemap of all DEGs clustered based on dissimilarity measures. (**C**) Clustering of eigen genes in the module. (**D**) Correlation between genes. (**E**) Heatmap of the correlation between modular feature genes and clinical features. Each unit corresponds to a correlation coefficient and a *P* value. (**F**) Scatter plot of module eigen genes in turquoise module.

The WGCNA method was used to find the overlapping genes that play an important part in the development of HCC modules and the key genes in the modules. Transcript expression profiles of 501 DEGs corresponding to overlapping genes in the TCGA database were selected and prepared for WGCNA analysis. On account of the gene expression pattern, the optimal soft threshold 4 found by the program was used as the soft threshold. The module selection criteria are cut height of 0.25 and minimum module size of 10. The clustering results show that the overlapping gene set is divided into eight modules. The correlation between the module and clinical trait data showed that the correlation between the turquoise module (correlation coefficient = 0.3, *P* = 1e−8) and Grade phenotype data was the most significant. There are 208 genes in the turquoise module (Figure 5). Eight key genes (TRM3 PPMIG PIGU RALY EFTUD2 PYGO2 STIP1 COPZ1) were obtained in the turquoise module (MM>0.8 and GS>0.2).

### GO enrichment and KEGG pathway analysis of genes in the turquoise module

**Figure 6:**
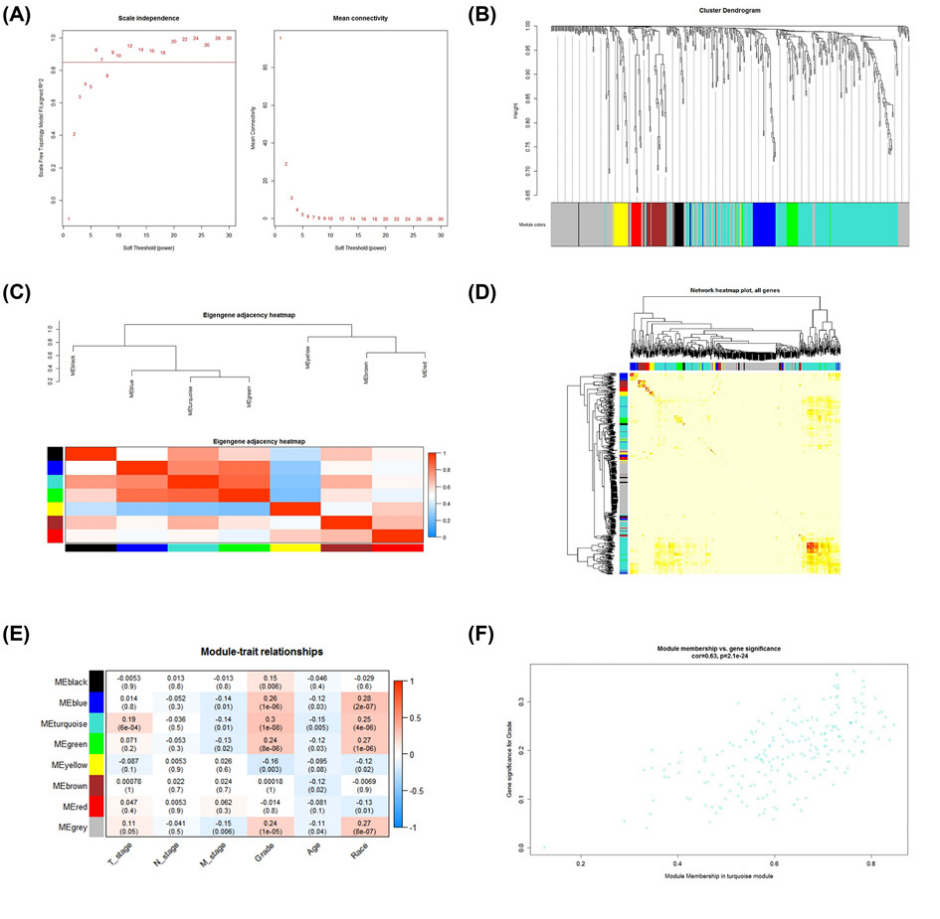
GO enrichment analysis chart, which is divided into biological process, CC and molecular function, and each part is arranged in descending order according to the first 10 terms of Count value (*P*<0.05)

**Figure 7:**
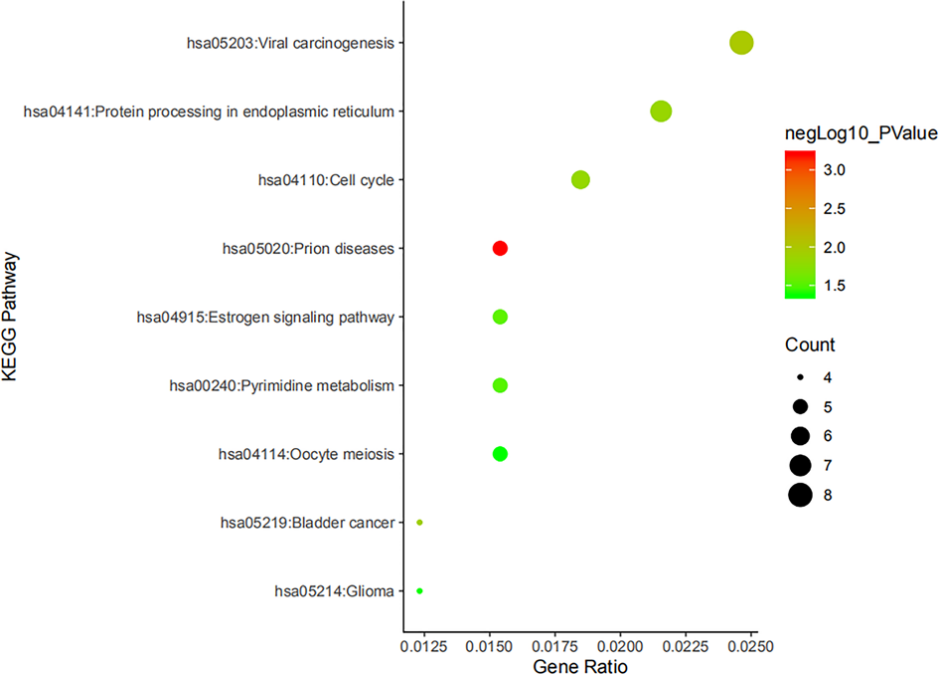
KEGG enrichment analysis The chart is arranged in descending order of Count value (*P*<0.05)

We perform GO analysis and KEGG pathway analysis on 208 genes in the turquoise module through the DAVID 6.8. Through GO analysis, 55 annotations were found to be enriched in BPs, cellular components (CCs) and MFs (Figure 6). GO analysis shows that in terms of BPs, genes are significantly enriched in certain cellular processes, such as cell division, mitotic nuclear division, etc. In terms of CCs, they are significantly enriched in the following cell groups: cytosol, extracellular exosome, etc. In terms of molecular function, genes are significantly enriched in protein binding (Table 2). KEGG pathway analysis yielded a total of nine enriched pathways (Figure 7). KEGG pathway analysis displays that the target genes were extensively involved in viral carcinogenesis, protein processing in endoplasmic reticulum and cell cycle (Table 3).

**Table 2:** GO enrichment analysis table (divided into GOTERM_BP_DIRECT, GOTERM_CC_DIRECT, GOTERM_CC_DIRECT three groups, *P*<0.05, each group is ranked according to the Count value from high to low, and the first 10 data of each group are taken)

Category	Term	Count	*P* value
GOTERM_BP_DIRECT	GO:0051301∼cell division	12	0.001766834
GOTERM_BP_DIRECT	GO:0007067∼mitotic nuclear division	10	0.001760521
GOTERM_BP_DIRECT	GO:0016032∼viral process	9	0.017941637
GOTERM_BP_DIRECT	GO:0000086∼G2/M transition of mitotic cell cycle	7	0.004120868
GOTERM_BP_DIRECT	GO:0006260∼DNA replication	7	0.007530052
GOTERM_BP_DIRECT	GO:0007049∼cell cycle	7	0.033261067
GOTERM_BP_DIRECT	GO:0000082∼G1/S transition of mitotic cell cycle	6	0.005413891
GOTERM_BP_DIRECT	GO:0007062∼sister chromatid cohesion	6	0.005640417
GOTERM_BP_DIRECT	GO:0000187∼activation of MAPK activity	6	0.00661362
GOTERM_BP_DIRECT	GO:0051726∼regulation of cell cycle	5	0.048344183
GOTERM_CC_DIRECT	GO:0005829∼cytosol	62	7.53E−06
GOTERM_CC_DIRECT	GO:0070062∼extracellular exosome	60	1.43E−07
GOTERM_CC_DIRECT	GO:0005654∼nucleoplasm	48	8.85E−04
GOTERM_CC_DIRECT	GO:0016020∼membrane	43	1.39E−04
GOTERM_CC_DIRECT	GO:0005789∼endoplasmic reticulum membrane	22	4.08E−04
GOTERM_CC_DIRECT	GO:0005783∼endoplasmic reticulum	20	0.001556487
GOTERM_CC_DIRECT	GO:0005925∼focal adhesion	10	0.025871509
GOTERM_CC_DIRECT	GO:0005913∼cell-cell adherens junction	9	0.023871692
GOTERM_CC_DIRECT	GO:0000790∼nuclear chromatin	8	0.004950709
GOTERM_CC_DIRECT	GO:0005765∼lysosomal membrane	8	0.029022075
GOTERM_MF_DIRECT	GO:0005515∼protein binding	139	3.87E−08
GOTERM_MF_DIRECT	GO:0005524∼ATP binding	26	0.036592117
GOTERM_MF_DIRECT	GO:0042802∼identical protein binding	16	0.026280507
GOTERM_MF_DIRECT	GO:0016301∼kinase activity	9	0.006798774
GOTERM_MF_DIRECT	GO:0098641∼cadherin binding involved in cell-cell adhesion	9	0.019123621
GOTERM_MF_DIRECT	GO:0001105∼RNA polymerase II transcription coactivator activity	4	0.008714107
GOTERM_MF_DIRECT	GO:0031492∼nucleosomal DNA binding	4	0.015792542
GOTERM_MF_DIRECT	GO:0046873∼metal ion transmembrane transporter activity	3	0.006750044
GOTERM_MF_DIRECT	GO:0005385∼zinc ion transmembrane transporter activity	3	0.026101256
GOTERM_MF_DIRECT	GO:0008475∼procollagen-lysine 5-dioxygenase activity	2	0.034083931

**Table 3:** KEGG path analysis table (ranked from highest to lowest according to the Count value)

Category	Term	Count	*P* value
KEGG_PATHWAY	hsa05203:Viral carcinogenesis	8	0.010157189
KEGG_PATHWAY	hsa04141:Protein processing in endoplasmic reticulum	7	0.014300805
KEGG_PATHWAY	hsa04110:Cell cycle	6	0.015123404
KEGG_PATHWAY	hsa05020:Prion diseases	5	6.32E−04
KEGG_PATHWAY	hsa04915:Estrogen signaling pathway	5	0.028902908
KEGG_PATHWAY	hsa00240:Pyrimidine metabolism	5	0.030805553
KEGG_PATHWAY	hsa04114:Oocyte meiosis	5	0.041413445
KEGG_PATHWAY	hsa05219:Bladder cancer	4	0.012156376
KEGG_PATHWAY	hsa05214:Glioma	4	0.040779829

### PPI network analysis of genes in Turquoise module

A total of 208 genes in the turquoise module to construct a PPI network by STRING. The nodes and lines in the figure represent genes and interactions between genes, respectively. The results of the PPI network obtained from STRING were imported into Cytoscape 3.6.1 for further visualization, and 42 genes were screened according to the degree of gene connectivity degree ≥8. The PPI network is shown in Supplementary Figure S3.

### Identification and validation of hub genes

**Figure 8:**
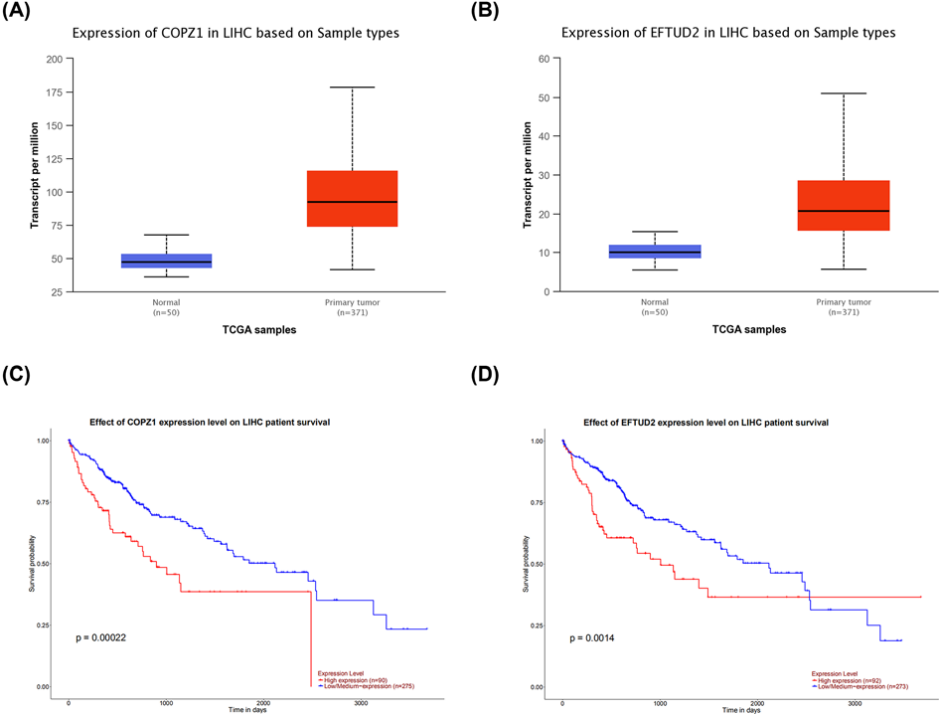
Box plot and survival curve of gene expression in HCC obtained by UALCAN (**A**) Differential expression of COPZ1 in normal tissues and liver cancer tissues (*P* = 1.624E−12). (**B**) Differential expression of EFTUD2 in normal tissues and liver cancer tissues (*P* = 1.625E−12). (**C**) The effect of differential expression of COPZ1 on survival rate of patients with liver cancer (*P* = 0.00022). (**D**) The effect of differential expression of EFTUD2 on survival rate of patients with liver cancer (*P* = 0.0014).

**Figure 9:**
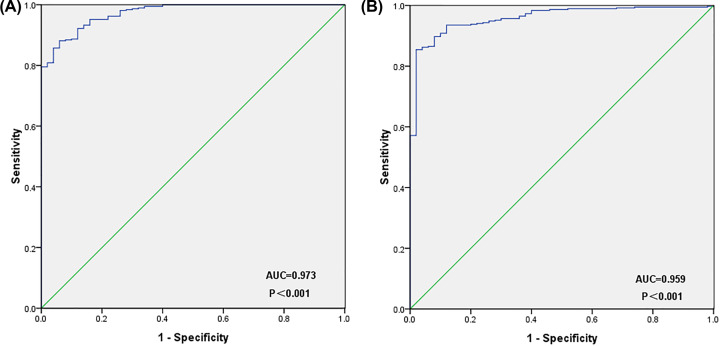
ROC curve of hub genes (**A**) COPZ1 (AUC = 0.973, *P*<0.001). (**B**) EFTUD2 (AUC = 0.959, *P*<0.001). Both COPZ1 and EFTUD2 have diagnostic significance (AUC>0.75) and statistical significance (*P*<0.05).

**Figure 10:**
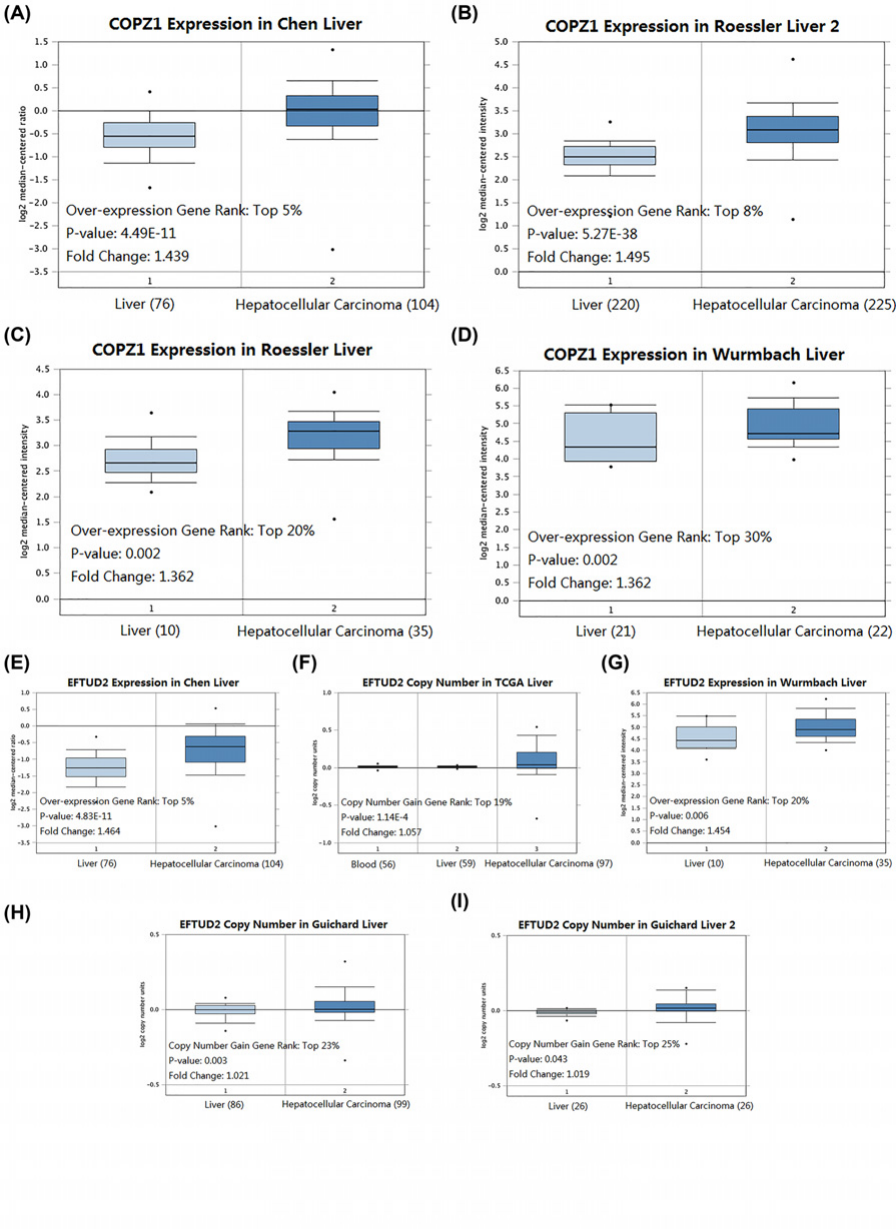
Gene expression in HCC and normal tissues from the Oncomine 4.5 database (**A**) Expression of COPZ1 in different databases: (**A**) Chen liver; (**B**) Roessler liver; 2 (**C**) Roessler liver; (**D**) Wurmbach liver. The selected databases were all up-regulated in HCC and had statistical significance (*P*<0.05). (**B**) Expressions of EFTUD2 in different databases: (**E**) Chen liver; (**F**) TCGA liver; (**G**) Wurmbach liver; (**H**) Guichard liver; (**I**) Guichard liver 2. Expressions of these genes were all up-regulated in HCC and had statistical significance for the selected databases (*P*<0.05).

The key genes of the significant module obtained by WGCNA above and the genes with the degree of connectivity of degree ≥8 obtained by PPI network analysis are intersected to obtain two hub genes, namely COPZ1 and EFTUD2. The Venn diagram is shown in Supplementary Figure S4. The expression of both genes in HCC tissues was higher than in normal tissues, as revealed by UALCAN. At the same time, they have significant prognostic significance for HCC and have high confidence (*P*<0.05) (Figure 8). ROC curves of COPZ1 (AUC = 0.973, *P*<0.001) and EFTUD2 (AUC = 0.959, *P*<0.001) were drawn from the TCGA database. COPZ1 and EFTUD2 have high diagnostic value (AUC>0.75), and both have statistical significance (*P*<0.05) (Figure 9). The gene expression and DNA copy number of the hub gene in HCC were obtained through the Oncomine 4.5 database, and data with high reliability were selected (*P*<0.05).The gene expression and DNA copy number of COPZ1 and EFTUD2 were significantly higher in liver cancer tissues than in normal tissues, and the Over-expression Gene Rank and DNA Copy Number Gain Gene Rank of all gene expressions are in the top 30% (Figure 10).The DNA methylation status of COPZ1 and EFTUD2 obtained from DiseaseMeth 2.0 in normal tissues was higher than that in cancer tissues (Supplementary Figure S5).

## Discussion

In the study, miR-331-3p was up-regulated in HCC from the GEO database. A large amount of data on miR-331-3p expression in HCC were collected by the GEO and TCGA databases. Then we performed meta-analysis based on GEO microarray and TCGA-based RNA-seq data to explore the diagnostic value of miR-331-3p in HCC. The prognostic significance of miR-331-3p in liver cancer is further confirmed by the study of the OS rate of HCC patients. We superimpose the 2206 HCC genes obtained by differential analysis of the TCGA database and the 6273 predicted target genes to obtain 501 overlapping genes. Important modules were further obtained through WGCNA analysis of overlapping genes, and eight key genes were screened out (TRM3 PPMIG PIGU RALY EFTUD2 PYGO2 STIP1 COPZ1). We performed PPI network analysis on genes in important modules, and selected 42 genes with a degree of connectivity of degree ≥8. The key genes in the important module and the genes with high gene connectivity obtained from the PPI network analysis are overlapped to obtain the hub gene (COPZ1 EFTUD2).

Although many studies have found that miR-331-3p is related to cancers such as colorectal cancer and, research on the regulation of miR-331-3p in HCC is still limited [40]. Chang et al. analyzed miRNA expression profiles and found that miR-331-3p can inhibit the expression of PH domain and leucine-rich repeat protein phosphatase (PHLPP) -mediated protein kinase B (AKT) and promote the proliferation of cancer cells [27]. Chen et al. found that miR-331-3p was significantly up-regulated in HCC through real-time PCR, which is consistent with our results [41]. As for miR-331-3p downstream genes, Cao et al. experimentally found that up-regulation of miR-331-3p inhibits the expression of inhibitor of growth family member 5 (ING5), and then promote the proliferation of HCC cells [42]. Cao et al. used qRT-PCR to find that HBV up-regulates the expression of miR-331-3p in HCC cell lines. And miR-331-3p directly inhibits VHL expression on 3′-UTR [29]. miR-331-3p was suggested as a possible prognostic marker in HCC [43]. Moreover miR-331-3p had been used as a critical marker for early detection of high-risk HCC patients [44]. Studies have shown that miR-331-3p has clinical significance in HCC and is considered a possible prognostic marker [45,46], which is consistent with the conclusions of our study. And the diagnostic potential of serum miR-331-3p has also been confirmed [47]. ROC curve obtained from the expression data of miR-331-3p in TCGA (AUC = 0.594, *P*<0.05) is shown in Supplementary Figure S6. It shows that miR-331-3p is of diagnostic significance.

Pathway analysis indicates that miR-331-3p is engaged in the inflammatory response of HCC through viral, endoplasmic reticulum and cell cycle pathways. Lots of investigations have shown that miR-331-3p is related to inflammatory process of HCC [48]. Results have shown that miR-331-3p is up-regulated by HBV and promotes HCC cell proliferation by inhibiting ING5 expression [42]. Recent experiments have shown that miR-331-3p can be a serum biomarker for early hepatitis C virus associated hepatocellular carcinoma [44]. The endoplasmic reticulum is an important organelle responsible for various functions, and there have been many studies on its relationship with cancer [49], including that endoplasmic reticulum stress is closely related to tumor treatment [50]. Research by Su et al. also verified that galangin inhibits HCC proliferation by inducing endoplasmic reticulum stress [51]. For the cell cycle, studies have shown that miR-138 inhibits HCC through the cyclin D3 (CCND3) gene regulating the cell cycle of HCC [52].

UALCAN was used to obtain COPZ1, EFTUD2 gene expression was up-regulated in HCC, and COPZ1, EFTUD2 have prognostic significance for HCC. The ROC curve shows that COPZ1 and EFTUD2 have an ideal diagnostic performance. Oncomine further confirmed that COPZ1 and EFTUD2 gene expression was up-regulated in HCC. The DNA methylation levels of COPZ1 and EFTUD2 obtained through DiseaseMeth were down-regulated in HCC, which is consistent with the results of the up-regulation in gene expression above. However, studies on COPZ1 and EFTUD2 in HCC are still few, but some studies have shown that COPZ1 and EFTUD2 are closely related to cancer development. COPZ1 is involved in inflammatory, intracellular traffic, autophagy and lipid homeostasis. And COPZ1 also shows some function in abortive autophagy, endoplasmic reticulum stress, unfolded protein response and cell apoptosis [53]. Research by Anania et al. demonstrates the key role of COPZ1 in thyroid tumor cell viability, suggesting that it may be considered an attractive target for new treatments for thyroid cancer [54]. Oliver's study elucidates the mechanism by which cancer cells emit apoptosis signals when COPZ1 is depleted. Depletion of COPZ1 can lead to loss of cancer-specific COPI function and subsequent paralysis of the Golgi apparatus. It also shows that COPZ1 is a significant target in the treatment of cancer [55]. EFTUD2, which is a spliceosome protein, was also involved in innate immunity and cell apoptosis [56]. EFTUD2 regulates RIG-I and MDA5 through mRNA splicing. EFTUD2 will be an interesting target to study the splicing mechanism by which EFTUD2 regulate on MyD88, RIG-I and MDA5 [57]. Studies by Sato et al. indicate that EFTUD2 plays an important part in the development of breast cancer. It is proved that depletion of SNW domain containing 1 (SNW1) and its related factor EFTUD2 can induce breast cancer cell apoptosis. In addition, expression of the SNW1 or EFTUD2 deletion construct can inhibit the association of endogenous proteins, thereby significantly increasing the number of apoptosis cells [58]. Later studies have shown that EFTUD2 accelerates the development of colitis-related tumors. EFTUD2 is constantly overexpressed in colon tissue and infiltrating macrophages. EFTUD2’s myeloid-specific knockout significantly inhibits chronic intestinal inflammation and tumorigenesis, which is related to the reduction in the production of inflammatory cytokines and tumorigenic factors [59]. Zhu et al. have shown that EFTUD2 mainly restricts HCV infection through a retinoic acid-inducible gene 1 (RIG-I)/melanoma differentiation-associated protein 5 (MDA5)-mediated pathway independent of JAK-STAT. And they suggested a potential antiviral pathway [60]. And experiments show that EFTUD2 inhibits HBV infection by up-regulating the expression of RIG-I [61], which is consistent with our description of the pathway above.

## Conclusion and perspectives

In the study, we comprehensively studied the role of miR-331-3p in HCC through weighted gene coexpression network analysis (WGCNA) based on TCGA, GEO and Oncomine. WGCNA were applied to build gene co-expression networks to examine the correlation between gene sets and clinical characteristics, and to identify hub genes and critical pathways. miR-331-3p is upregulated in HCC and demonstrates good prognosis and diagnostic performance for HCC based on the GEO and TCGA data sets. HCC-related genes obtained from the TCGA database overlapped with miR-331-3p potential genes, and 501 target genes for miR-331-3p related to HCC were obtained. Based on overlapping genes, the critical turquoise module and its eight key genes were screened by WGCNA. Genes in the turquoise module were analyzed for enrichment analysis to explore their role in the BPs of HCC. Based on the genes in the turquoise module, 48 genes with the degree of gene connectivity ≥8 were obtained by PPI analysis. Both genes (COPZ1 EFTUD2) were obtained by overlapping the key genes and those obtained by PPI analysis. The relationship between miR-331-3p targeting COPZ1 and EFTUD2 and HCC was explored from gene expression and DNA methylation. The study of miR-331-3p may be helpful for understanding the genetic level of HCC progression and revealing its potential molecular mechanisms and regulatory networks.

There are still some problems to be solved in the study. First of all, the data of existing databases for bioinformatic analysis and data mining were applied in the study. We hope to verify our conclusions experimentally in the future work. Second, there are few studies on the role of COPZ1 and EFTUD2 in HCC. The role of both genes in HCC was required to be studied in detail. Third, the diagnostic performance of miRNA-331-3p could be better presented if the expression level from serum samples was determined of HCC and analyzed. Fourth, the infiltration of immune cells may be related to the progress of HCC. Taking immune infiltration into consideration will help us to further explore the role of miR-331-3p in HCC.

## Supplementary Material

Supplementary Figures S1-S6 and Table S1

## Abbreviations

AUC: area under curve
BP: biological process
CC: cellular component
CCND3: cyclin D3
CI: confidence interval
COPZ1: coatomer protein complex subunit zeta 1
DEG: differentially expressed gene
DEM: differentially expressed microRNA
EFTUD2: elongation factor Tu GTP binding domain containing 2
EMT: epithelial mesenchymal transition
GEO: Gene Expression Omnibus
GO: gene ontology
GS: gene significance
HBV: hepatitis B virus
HCC: hepatocellular carcinoma
HCV: hepatitis C virus
ING5: inhibitor of growth family member 5
KEGG: Kyoto, Encyclopedia of Genes and Genomes
MDA5: melanoma differentiation-associated protein 5
ME: module eigengene
MF: molecular functions
miRNA: microRNA
MM: module membership
PC: pancreatic cancer
PPI: protein–protein interaction
RIG-I: retinoic acid-inducible gene 1
ROC: receiver operating characteristic
SNW1: SNW domain containing 1
SMD: standard mean difference
ST7L: suppression of tumorigenicity 7 like
TCGA: The Cancer Genome Atlas
VHL: von Hippel–Lindau tumor suppressor
WGCNA: weighted gene coexpression network analysis
