# Supplementary material for: Potential targets and molecular mechanism of miR-331-3p in hepatocellular carcinoma identified by weighted gene coexpression network analysis
Source: Biosci Rep. 2020 Jun 25;40(6):BSR20200124. doi: 10.1042/BSR20200124 (PMC7317601; doi:10.1042/BSR20200124)
Supplement: Supplementary Figures S1-S6 and Table S1 [file BSR-2020-0124_supp.pdf]

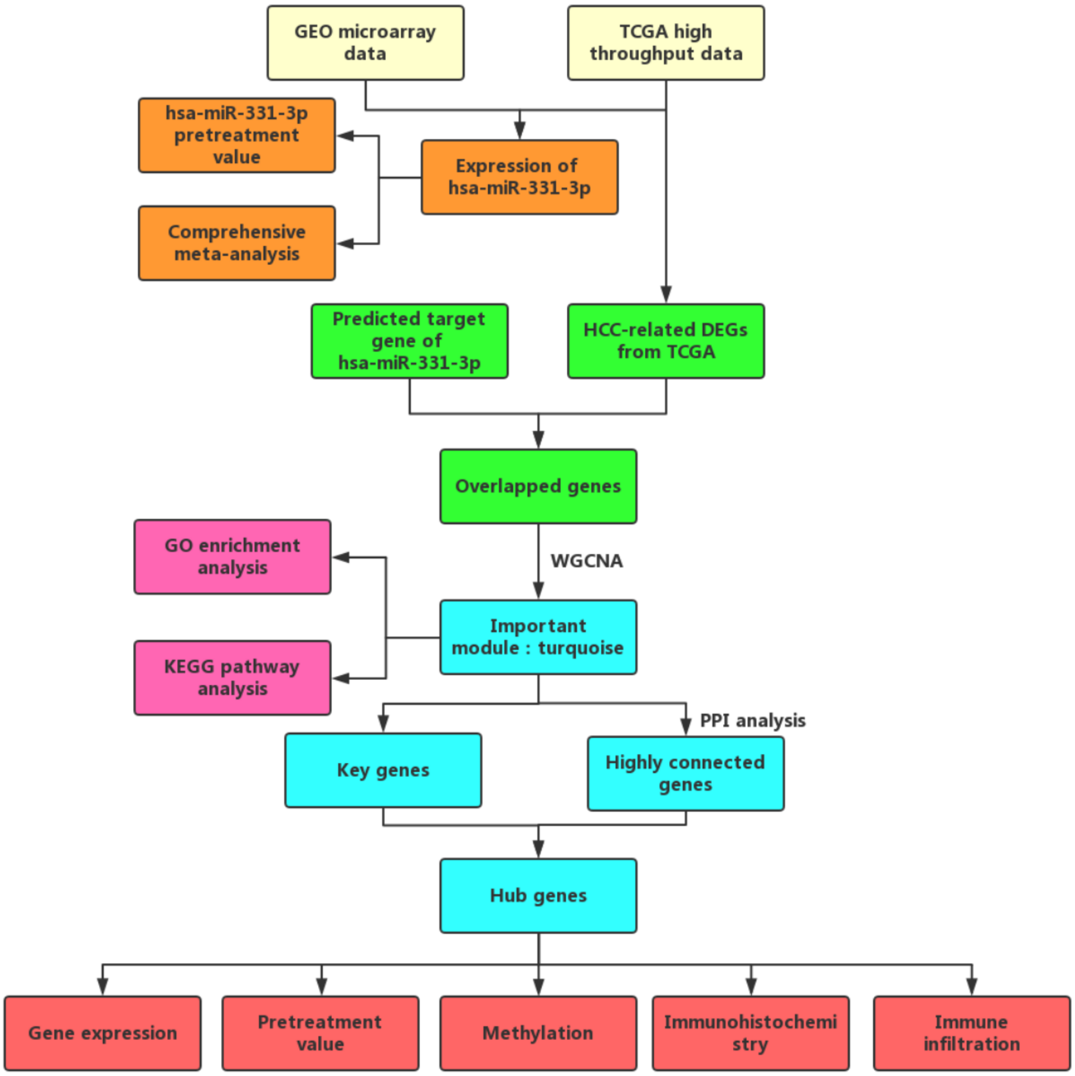

FigureS1. Work flow diagram.

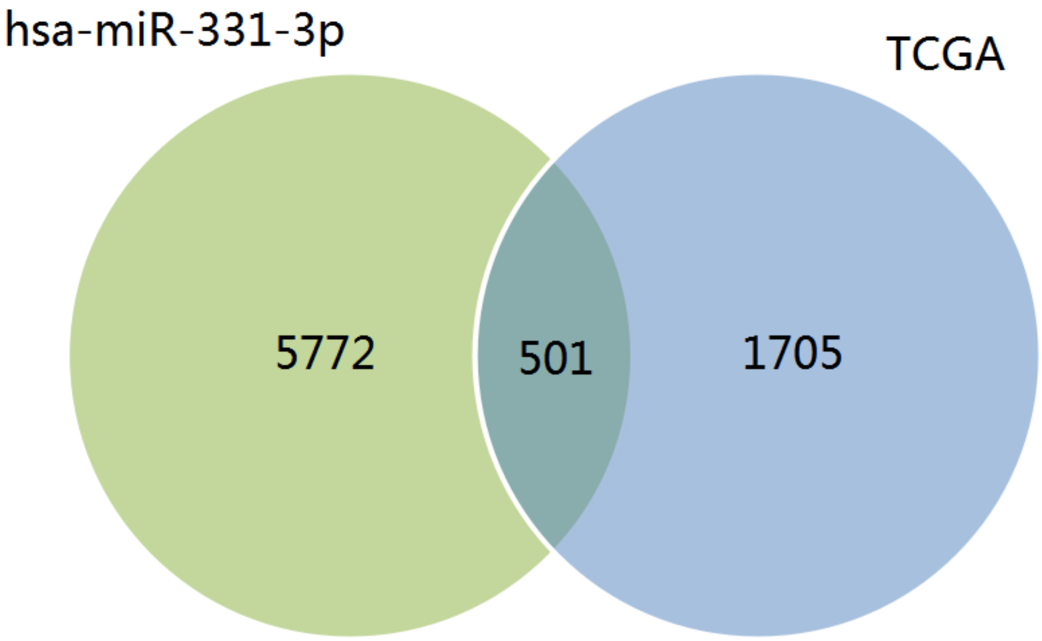

FigureS2. Venn plot of overlapping gene overlaps of miR-331-3p predicted by HCC-related DEGs and miRwalk2.0 in TCGA. Green: the miR-331-3p overlapping gene predicted by miRwalk2.0; blue :HCC-related DEGs in TCGA.Finally, 501 overlapping genes were obtained.

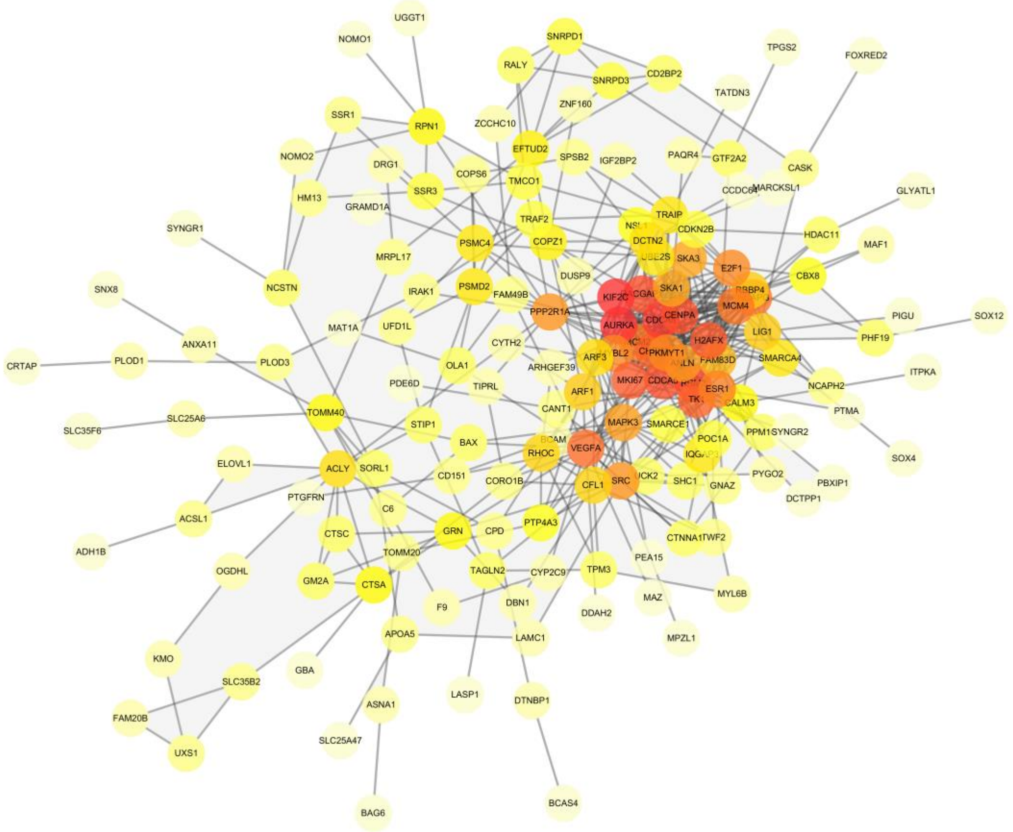

FigureS3. PPI network visualized by Cytoscape. The color of the node changes from yellow to red according to the Degree value.

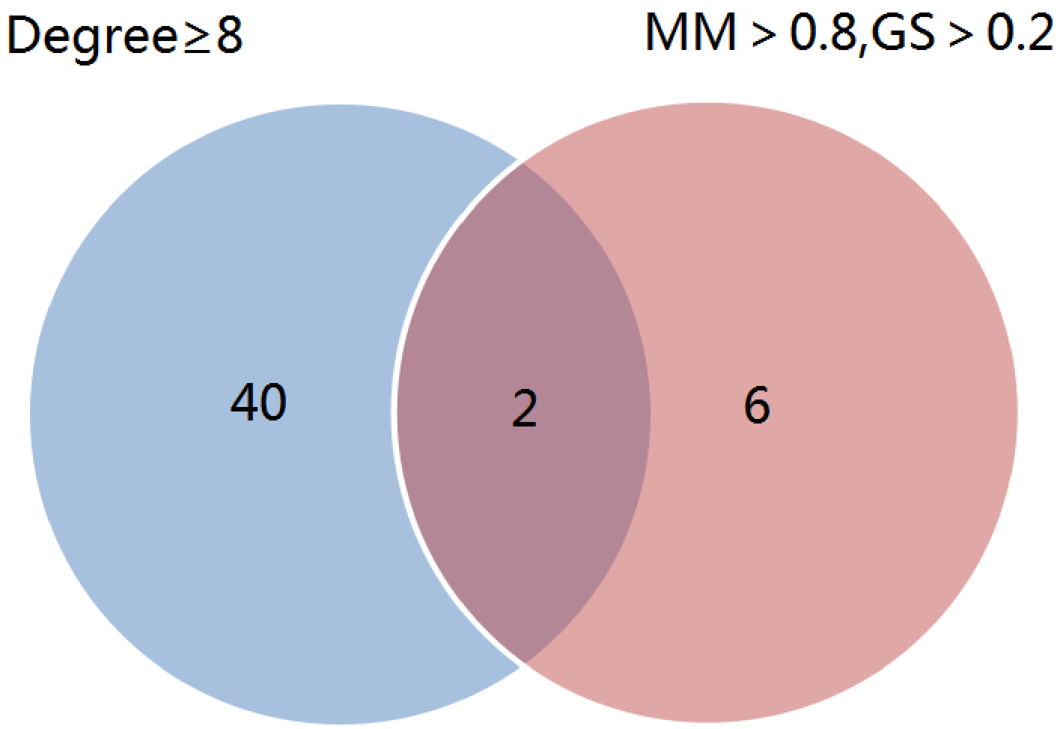

FigureS4. Venn plot for hub genes. Hub genes were obtained from target genes in important modules (Module Membership> 0.8, Gene Significance> 0.2) and PPI analysis (Degree ≥ 8) of genes in important modules. Blue: PPI analysis (Degree ≥ 8) of genes in important modules; red: the target genes in important modules (Module Membership> 0.8, Gene Significance> 0.2).

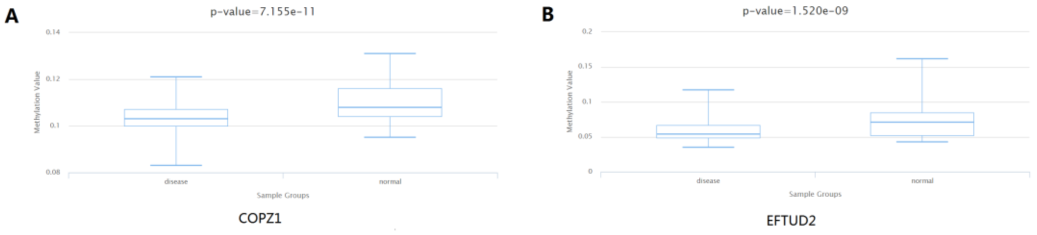

FigureS5. Methylation analysis of the hub gene. The methylation levels of A.COPZ1 (p = 7.155e-11) and B.EFTUD2 (p = 1.520e-09) in HCC and normal tissues surrounding the tumor were obtained using DiseaseMeth 2.0.

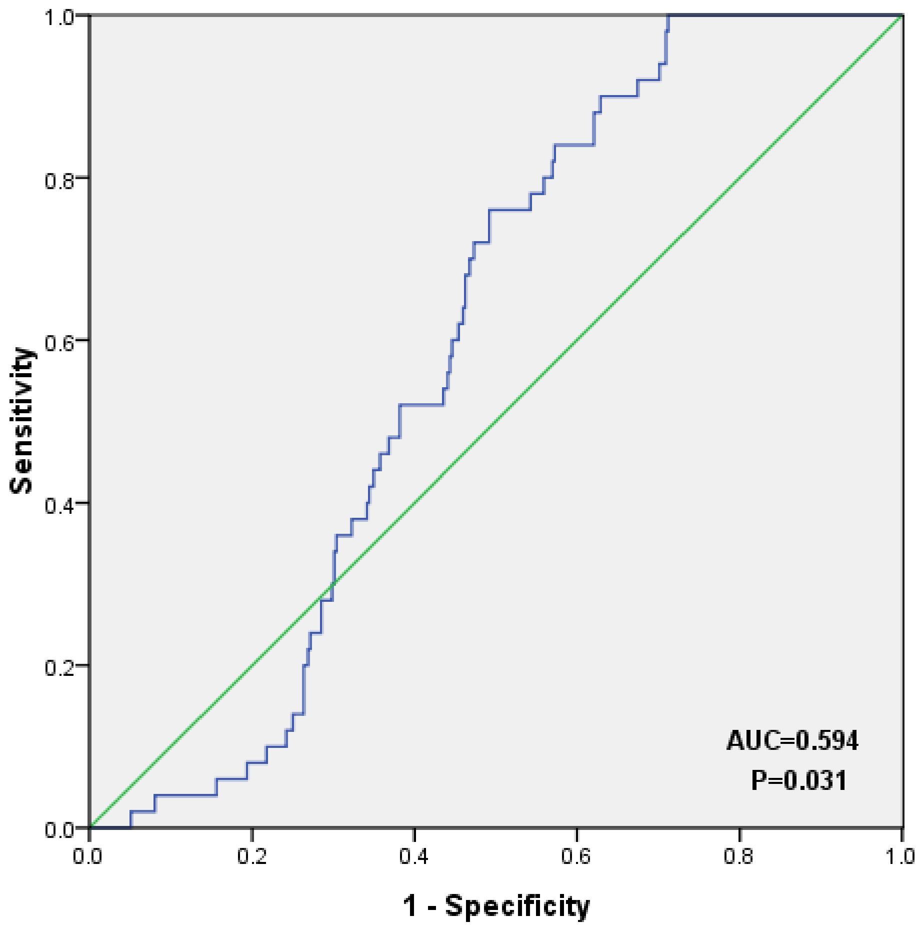

FigureS6.ROC curve of miR-331-3p (AUC=0.594 , $p < 0.05$).

Table S1. Overall Survival (OS) ，yearofformcompletion ，yearofinitialpathologicdiagnosis of HCC patients in the TCGA database.

	OS	Yearofformcompletion	Yearofinitialpathologicdiagnosis
TCGA-2Y-A9GT	1624	2014	2006
TCGA-BC-A10U	837	2011	2000
TCGA-2Y-A9GU	1939	2014	2009
TCGA-CC-A3MC	363	2012	2011
TCGA-G3-A25X	1779	2011	2007
TCGA-G3-A7M8	430	2014	2013
TCGA-2Y-A9GW	1271	2014	2007
TCGA-DD-AACM	1769	2014	2009
TCGA-DD-A39X	1694	2012	1998
TCGA-MR-A8JO	330	2013	2013
TCGA-ZP-A9CZ	706	2014	2011
TCGA-DD-AAD3	1295	2014	2011
TCGA-ZS-A9CF	2412	2014	2008
TCGA-G3-A7M6	632	2014	2013
TCGA-2Y-A9H5	555	2014	2010
TCGA-DD-AAE2	638	2014	2013
TCGA-XR-A8TF	693	2015	2012
TCGA-G3-AAV7	361	2014	2013
TCGA-5R-AA1D	449	2014	2013
TCGA-G3-A25S	416	2011	2009
TCGA-DD-AACE	2184	2014	2008
TCGA-WX-AA46	756	2014	2012
TCGA-XR-A8TG	898	2015	2012
TCGA-ED-A66Y	296	2013	2013
TCGA-DD-A115	2542	2010	2002
TCGA-LG-A9QC	425	2014	2013
TCGA-DD-A73E	44	2013	2012

TCGA-RC-A7SH	468	2014	2012
TCGA-EP-A12J	570	2011	2010
TCGA-UB-A7MF	214	2014	2013
TCGA-2Y-A9H9	697	2014	2011
TCGA-5C-A9VH	322	2014	2013
TCGA-G3-A25V	860	2011	2010
TCGA-ED-A66X	406	2013	2013
TCGA-DD-AADV	574	2014	2013
TCGA-DD-AAW2	1855	2014	2009
TCGA-WQ-AB4B	395	2014	2013
TCGA-ED-A7PX	6	2014	2013
TCGA-DD-A4NJ	928	2013	2011
TCGA-ZP-A9D4	395	2014	2012
TCGA-BC-A110	2116	2011	2003
TCGA-2Y-A9GY	757	2014	2007
TCGA-DD-AADO	453	2014	2013
TCGA-DD-A1EG	1372	2013	2007
TCGA-DD-AAE0	555	2014	2013
TCGA-DD-A4NB	989	2012	2011
TCGA-DD-AADK	1049	2014	2011
TCGA-MI-A75G	698	2013	2012
TCGA-FV-A2QR	581	2012	2006
TCGA-DD-AAE7	644	2014	2012
TCGA-2Y-A9H7	1168	2014	2011
TCGA-CC-A5UE	272	2014	2012
TCGA-K7-A5RG	519	2013	2012
TCGA-ED-A4XI	819	2013	2012
TCGA-RC-A7S9	640	2014	2012
TCGA-DD-AACY	1450	2014	2010
TCGA-FV-A3I1	247	2012	2011
TCGA-DD-A11C	662	2010	2006
TCGA-DD-AADD	1231	2014	2011

TCGA-FV-A496	10	2013	2012
TCGA-DD-A3A1	233	2012	2001
TCGA-DD-A4NE	660	2012	2011
TCGA-MI-A75E	507	2013	2013
TCGA-2Y-A9H0	3675	2014	2004
TCGA-2Y-A9H2	1731	2014	2010
TCGA-BC-A217	1397	2011	2010
TCGA-DD-AADG	1145	2014	2011
TCGA-DD-AACG	469	2014	2008
TCGA-DD-AACK	9	2014	2008
TCGA-NI-A8LF	799	2014	2012
TCGA-DD-AAD1	564	2014	2010
TCGA-DD-AADN	898	2014	2012
TCGA-ZP-A9CY	782	2014	2011
TCGA-G3-A5SJ	698	2013	2012
TCGA-BC-A3KF	8	2012	2011
TCGA-FV-A3I0	848	2012	2011
TCGA-DD-AAEB	478	2014	2012
TCGA-2Y-A9GZ	848	2014	2007
TCGA-CC-A8HS	300	2014	2013
TCGA-DD-A1EB	2017	2011	2001
TCGA-DD-AADY	555	2014	2013
TCGA-DD-AAVP	2752	2014	2007
TCGA-G3-A5SK	744	2013	2012
TCGA-RC-A7SF	579	2014	2012
TCGA-G3-AAV3	412	2014	2013
TCGA-KR-A7K8	906	2014	2012
TCGA-ED-A627	423	2013	2012
TCGA-BC-A10Z	34	2011	2003
TCGA-EP-A3RK	363	2012	2012
TCGA-UB-A7ME	486	2014	2013
TCGA-ED-A7XP	400	2013	2013

TCGA-UB-A7MA	848	2014	2013
TCGA-5R-AAAM	46	2014	2013
TCGA-DD-AACP	415	2014	2009
TCGA-FV-A2QQ	729	2012	2011
TCGA-DD-AACJ	2102	2014	2008
TCGA-HP-A5MZ	91	2013	Not Available
TCGA-CC-A8HT	140	2014	2013
TCGA-CC-5259	250	2012	2010
TCGA-DD-A4NK	1210	2013	2004
TCGA-RC-A7SK	472	2014	2013
TCGA-DD-AAEE	810	2014	2012
TCGA-YA-A8S7	412	2014	2012
TCGA-BC-A10W	91	2011	2002
TCGA-CC-A5UC	347	2014	2012
TCGA-DD-A1EI	183	2011	2008
TCGA-DD-AAD2	658	2014	2010
TCGA-DD-AAD6	672	2014	2011
TCGA-XR-A8TC	1339	2015	2011
TCGA-UB-A7MB	601	2014	2013
TCGA-DD-A4NA	1008	2012	2009
TCGA-DD-A4NG	802	2013	2011
TCGA-2Y-A9GV	2532	2014	2007
TCGA-DD-AAD0	137	2014	2010
TCGA-DD-AAE9	722	2014	2012
TCGA-BC-4073	849	2010	2009
TCGA-DD-AACF	365	2014	2008
TCGA-DD-A11A	79	2010	2004
TCGA-DD-A73A	728	2013	2012
TCGA-4R-AA8I	262	2014	2013
TCGA-DD-AAW0	2015	2014	2009
TCGA-BW-A5NP	0	2014	2010
TCGA-DD-A3A2	2131	2012	1998

TCGA-DD-A4NO	2245	2013	2007
TCGA-KR-A7K2	829	2014	2013
TCGA-BC-A10Y	711	2011	2002
TCGA-CC-A9FW	248	2014	2013
TCGA-DD-AACA	2301	2014	2008
TCGA-ZP-A9D0	1091	2014	2011
TCGA-PD-A5DF	639	2013	2007
TCGA-DD-AAVS	1823	2014	2007
TCGA-G3-A7M5	447	2014	2013
TCGA-DD-A1E9	2759	2011	2002
TCGA-CC-A123	219	2011	2010
TCGA-G3-AAV1	359	2014	2013
TCGA-BC-A69H	444	2013	2013
TCGA-BC-A3KG	680	2012	2011
TCGA-DD-AAW1	1989	2014	2009
TCGA-WX-AA44	615	2014	2011
TCGA-DD-AACH	195	2014	2008
TCGA-DD-A73B	283	2013	2012
TCGA-DD-AAW3	1633	2014	2009
TCGA-DD-AAVV	2455	2014	2007
TCGA-RC-A6M4	22	2015	2010
TCGA-RC-A7SB	588	2014	2012
TCGA-BW-A5NO	20	2014	2010
TCGA-DD-A73C	701	2013	2012
TCGA-DD-AADA	1233	2014	2011
TCGA-FV-A4ZQ	12	2013	2012
TCGA-2Y-A9H4	1452	2014	2010
TCGA-DD-AADB	1242	2014	2011
TCGA-DD-AACL	107	2014	2008
TCGA-DD-AAEK	1067	2014	2010
TCGA-DD-A73G	3478	2013	2005
TCGA-DD-AACS	1804	2014	2009

TCGA-G3-AAV2	372	2014	2013
TCGA-DD-AADS	474	2014	2013
TCGA-BC-A5W4	547	2013	2012
TCGA-MR-A520	229	2012	2012
TCGA-XR-A8TE	925	2015	2012
TCGA-DD-A114	1149	2010	2005
TCGA-DD-AAVR	2513	2014	2007
TCGA-RC-A6M3	0	2015	2009
TCGA-3K-AAZ8	396	2014	2013
TCGA-G3-A5SM	520	2013	2012
TCGA-O8-A75V	538	2013	2013
TCGA-ZS-A9CD	1386	2014	2010
TCGA-2Y-A9H1	1229	2014	2010
TCGA-2Y-A9HB	260	2014	2012
TCGA-DD-AACO	1876	2014	2009
TCGA-G3-AAV5	354	2014	2013
TCGA-LG-A9QD	366	2014	2013
TCGA-ZP-A9D2	765	2014	2012
TCGA-DD-AAVW	2317	2014	2008
TCGA-DD-AAEI	1531	2014	2010
TCGA-CC-A9FU	0	2014	2013
TCGA-DD-AACZ	171	2014	2010
TCGA-5C-AAPD	20	2014	2013
TCGA-2Y-A9H8	633	2014	2011
TCGA-DD-AA3A	410	2014	2009
TCGA-BD-A2L6	1363	2012	2011
TCGA-DD-AADM	12	2014	2012
TCGA-DD-A3A3	535	2012	1999
TCGA-DD-A1EJ	1005	2011	2008
TCGA-UB-AA0V	314	2014	2013
TCGA-DD-AAC8	16	2014	2008
TCGA-DD-A3A4	612	2012	1998

TCGA-GJ-A6C0	31	2013	2012
TCGA-CC-A7II	399	2014	2013
TCGA-EP-A3JL	303	2012	2011
TCGA-DD-A73D	693	2013	2012
TCGA-BC-A10R	308	2011	1999
TCGA-DD-AADL	636	2014	2012
TCGA-BW-A5NQ	0	2014	2010
TCGA-DD-AAC9	347	2014	2008
TCGA-DD-A4NQ	373	2013	2007
TCGA-G3-A7M9	56	2014	2013
TCGA-DD-A4ND	2746	2012	2007
TCGA-5C-A9VG	328	2014	2013
TCGA-5R-AA1C	520	2014	2013
TCGA-ED-A82E	408	2014	2013
TCGA-G3-AAV4	27	2014	2012
TCGA-CC-A7IK	262	2014	2013
TCGA-G3-A3CK	585	2012	2011
TCGA-CC-A8HV	279	2014	2013
TCGA-BC-A10T	837	2011	2000
TCGA-G3-A3CG	673	2012	2011
TCGA-DD-AACT	1562	2014	2010
TCGA-DD-A73F	1085	2013	2010
TCGA-G3-A25Z	655	2011	2010
TCGA-CC-A3MB	315	2012	2011
TCGA-DD-A1EK	558	2013	2008
TCGA-DD-A116	1622	2010	2002
TCGA-DD-AAE1	552	2014	2013
TCGA-DD-A1EL	415	2011	2009
TCGA-2Y-A9H3	1516	2014	2010
TCGA-DD-AADJ	1066	2014	2011
TCGA-RG-A7D4	1098	2014	2012
TCGA-DD-AAVU	2202	2014	2008

TCGA-G3-A25W	935	2011	2010
TCGA-K7-A5RF	631	2013	2012
TCGA-G3-A7M7	361	2014	2013
TCGA-BD-A3ER	1115	2012	2011
TCGA-FV-A3R3	366	2012	2012
TCGA-DD-AAD8	1219	2014	2011
TCGA-UB-AA0U	327	2014	2013
TCGA-CC-5262	103	2011	2010
TCGA-CC-A5UD	304	2014	2012
TCGA-DD-AACV	1531	2014	2010
TCGA-CC-A8HU	344	2014	2013
TCGA-MI-A75C	291	2013	2012
TCGA-DD-A4NP	3308	2013	2005
TCGA-MI-A75I	630	2013	2011
TCGA-MI-A75H	747	2013	2012
TCGA-DD-AACU	1567	2014	2010
TCGA-ED-A459	910	2013	2012
TCGA-BC-4072	1490	2010	2005
TCGA-BC-A8YO	562	2014	2013
TCGA-DD-AADF	115	2014	2011
TCGA-DD-A119	223	2010	2003
TCGA-DD-A4NI	816	2013	2011
TCGA-WQ-A9G7	30	2014	2013
TCGA-G3-AAUZ	480	2014	2013
TCGA-DD-AACW	1424	2014	2010
TCGA-G3-A25Y	452	2011	2006
TCGA-FV-A23B	1852	2011	2005
TCGA-DD-AACB	2324	2014	2008
TCGA-ZP-A9D1	21	2014	2012
TCGA-LG-A6GG	387	2013	2013
TCGA-DD-AACD	381	2014	2008
TCGA-G3-A3CJ	594	2012	2011

TCGA-G3-A5SL	621	2013	2012
TCGA-ED-A5KG	854	2013	2012
TCGA-KR-A7K0	65	2014	2013
TCGA-DD-AADE	1202	2014	2011
TCGA-CC-A7IE	217	2014	2012
TCGA-XR-A8TD	1030	2015	2012
TCGA-2Y-A9H6	357	2014	2011
TCGA-BC-A69I	387	2013	2013
TCGA-DD-A39V	643	2012	2000
TCGA-DD-AAVQ	2728	2014	2007
TCGA-ED-A8O6	56	2014	2013
TCGA-G3-A3CI	180	2012	2011
TCGA-CC-5261	97	2011	2010
TCGA-EP-A2KB	596	2011	2011
TCGA-RC-A6M5	15	2015	2012
TCGA-DD-A11D	1560	2010	2006
TCGA-G3-A25U	1636	2011	2007
TCGA-G3-A25T	1553	2011	2007
TCGA-KR-A7K7	951	2014	2012
TCGA-DD-AADU	554	2014	2013
TCGA-WX-AA47	556	2014	2012
TCGA-DD-A39Y	171	2012	2002
TCGA-DD-AAD5	1345	2014	2011
TCGA-DD-A113	2425	2010	2006
TCGA-EP-A2KC	19	2011	2011
TCGA-DD-A1EF	394	2011	2007
TCGA-DD-AACC	1685	2014	2008
TCGA-ES-A2HS	688	2011	2008
TCGA-CC-A7IL	278	2014	2013
TCGA-DD-A3A9	931	2012	2001
TCGA-DD-AACI	1618	2014	2008
TCGA-DD-AAVY	1970	2014	2009

TCGA-DD-AAVX	1718	2014	2008
TCGA-RC-A6M6	9	2015	2011
TCGA-CC-A9FS	211	2014	2013
TCGA-G3-AAV6	65	2014	2013
TCGA-CC-5264	102	2011	2010
TCGA-NI-A4U2	1791	2013	2005
TCGA-CC-5263	129	2011	2010
TCGA-K7-A6G5	512	2013	2013
TCGA-DD-A39W	827	2012	1995
TCGA-CC-A7IJ	382	2014	2013
TCGA-BC-A10Q	1135	2011	1998
TCGA-ZP-A9CV	1088	2014	2011
TCGA-ZS-A9CG	341	2014	2013
TCGA-DD-A3A6	3258	2012	1997
TCGA-DD-AACQ	432	2014	2009
TCGA-DD-A4NN	899	2013	2004
TCGA-ED-A7PZ	6	2014	2013
TCGA-DD-AAVZ	1900	2014	2009
TCGA-WJ-A86L	345	2014	2013
TCGA-FV-A495	1	2013	2012
TCGA-DD-AAE6	141	2014	2012
TCGA-GJ-A9DB	67	2014	2013
TCGA-BC-A10S	1423	2011	1999
TCGA-DD-AADR	2028	2014	2009
TCGA-DD-A3A7	419	2013	1998
TCGA-CC-A7IF	649	2014	2013
TCGA-CC-A9FV	0	2014	2013
TCGA-DD-AAED	763	2014	2012
TCGA-DD-AADQ	436	2014	2013
TCGA-DD-AAE4	608	2014	2013
TCGA-GJ-A3OU	879	2015	2011
TCGA-BC-A216	1351	2011	2008

TCGA-BC-A112	153	2011	2009
TCGA-FV-A3R2	194	2012	2005
TCGA-DD-A4NR	9	2013	2005
TCGA-G3-A5SI	768	2013	2011
TCGA-DD-A39Z	601	2012	1996
TCGA-CC-5258	129	2011	2010
TCGA-DD-A3A8	11	2012	2001
TCGA-DD-AADI	1085	2014	2011
TCGA-DD-A3A5	3125	2012	1996
TCGA-DD-AACX	170	2014	2010
TCGA-2Y-A9HA	36	2014	2012
TCGA-DD-AADW	587	2014	2013
TCGA-K7-AAU7	359	2014	2013
TCGA-BC-A10X	770	2011	2002
TCGA-DD-AADC	425	2014	2011
TCGA-EP-A26S	608	2011	2011
TCGA-DD-AACN	1302	2014	2009
TCGA-DD-AAEA	575	2014	2012
TCGA-CC-A3M9	300	2012	2011
TCGA-DD-A11B	14	2010	2005
TCGA-DD-A4NF	942	2013	2011
TCGA-DD-A1EH	1495	2011	2008
TCGA-CC-5260	87	2011	2010
TCGA-ED-A7XO	427	2013	2013
TCGA-2Y-A9GX	2442	2014	2007
TCGA-DD-AAEG	719	2014	2012
TCGA-CC-A3MA	303	2012	2011
TCGA-ED-A8O5	406	2014	2013
TCGA-CC-A7IH	365	2014	2013
TCGA-QA-A7B7	94	2013	2013
TCGA-ES-A2HT	438	2011	2007
TCGA-DD-A3A0	785	2012	2001

TCGA-G3-A3CH	780	2012	2010
TCGA-UB-A7MD	52	2014	2013
TCGA-DD-AADP	458	2014	2013
TCGA-DD-AAEH	784	2014	2012
TCGA-DD-A1EA	2415	2011	2002
TCGA-DD-A4NH	917	2013	2011
TCGA-DD-A1EE	349	2011	2006
TCGA-DD-A1ED	2301	2011	2006
TCGA-DD-A4NL	1711	2013	2007
TCGA-HP-A5N0	752	2014	Not Available
TCGA-2Y-A9GS	724	2014	2006
TCGA-BD-A3EP	409	2012	2010
TCGA-G3-AAV0	476	2014	2013
TCGA-DD-A4NS	2456	2013	2005
TCGA-DD-A118	3437	2010	2003
TCGA-EP-A2KA	627	2011	2011
TCGA-ZS-A9CE	1241	2014	2011
TCGA-ED-A7PY	390	2014	2013
TCGA-G3-A6UC	671	2013	2012
TCGA-FV-A4ZP	2486	2013	2005
TCGA-UB-A7MC	500	2014	2013
TCGA-DD-A1EC	602	2011	2004
TCGA-T1-A6J8	23	2014	2013
TCGA-DD-AAE8	664	2014	2012
TCGA-DD-A4NV	2398	2013	2007
TCGA-CC-A7IG	299	2014	2013
TCGA-DD-AAE3	566	2014	2013
TCGA-CC-A1HT	101	2011	2010
TCGA-ED-A97K	6	2014	2013
